# From Our Correspondents

**Published:** 1987-11

**Authors:** 


### From Our Correspondents

### Bristol Hosts BMA Parliament

Bristol '67. Bristol '87. Bristol '07? How will the BMA change before the next Annual Representative Meeting in Bristol, if the 20 year interval between hosting meetings is repeated? How many of us will be there? Will seasoned representatives looking back on '87 sigh as nostalgically as we now do about '67? How many of us could have foreseen in '67 that the BMA would now be a statutory Trade Union (by summary Act of another Parliament); that the profession would now directly elect a majority of members on a reformed GMC; or that women would have won equality with men in admission to medical schools?

Inevitably there is change. Reassuringly, there is also continuity. Robert Cooke's presidential mantle of '67 fell upon the shoulders of fellow surgeon and Bristol graduate David Bolt. Familiar faces and reliable pairs of hands, led by John and Jill Mitchell, discharged the duties of the local organising committee with predictable aplomb and to general acclaim.

The 500 plus "reps", plus accompanying persons (no longer necessarily 'ladies') seek different satisfactions from their five day stint; a change of air, relaxation with friends old and new, sustenance for the inner person, a spot of golf, stimulating debates and, who knows, perhaps even a hand in making history. At best, what they find is a fortuitous mix of enjoyment and endurance.

At a healthy temporal distance, what highlights spring to mind? Town and Gown alike did us proud. A happy tradition dictates that the first act of the play should be an ecumenical service, and the curtain rose on an expectant cathedral; pomp and piety joined hands to the soaring strains of the organ and Juliet Booth's glorious voice in Mozart's Jubilate. Centre stage, my Lord Bishop, the Rt. Rev. Barry Rogerson, set the scene for the acts to follow with a ringing demand for equality in health care in the name of a loving God and a just society. We then repaired to Ashton Court to endorse his sentiments as we enjoyed the fruits of our own advantages in life, with more Mozart and 'A Little Night Music' from the strings of the Southmead Orchestra.

The following morning the incongruously named Holiday Inn witnessed the transition from the sacred to the secular. The Lord Mayor, Councillor Marmaduke Alderson, as always a resplendent figure, invited bemused reps to acknowledge the benefactions bestowed upon their host city by the benighted tobacco manufacturing industry, adding with a true professional's timing, "I am a non-smoker." The climax of the play was, as always, the adjourned Annual General Meeting, for which the Great Hall, forever evocative of Winston Churchill's chancellorship, was the setting. To the peals of another splendid organ – the Bristol Sound – the Vice Chancellor, Sir John Kingman, led the University procession to hear David Bolt's sobering presidential address in which he reviewed continuity and change in the GMC's Conduct Committee work, over which he also presides. The City Museum's lavish portions of strawberries and cream (remember the iced coffee laced with rum in '67?) were a welcome antidote.

Visits charmed and educated in varying measure. John Clamp's scientific meeting broke new ground with the inspired idea of holding sessions in places of intrinsic medical interest such as Jenner's temple of vaccinia and Oldbury Nuclear Power station. "Tribal" dinners catered for those who know that "wha's like us, damned few, and they're a'deid". The reps' dinner dance, betimes a duty, was made a pleasure by Eric Dehn's hilarious speech.

What of the meeting itself? Chairmen duly reported upon the stewardship of their diligent committee. Reps duly passed judgement and elected those who would serve and lead them until they met again. Policy was duly made upon the usual bewildering range of business, concerned predominantly with ethical and scientific issues and assisted (or impeded?) by a record number of nearly 1000 motions. What masochists the Agenda Committee are, as I can testify from 16 years' service. As always, the unexpected was to be expected. We should have foreseen that the meeting would affirm against the advice of the "establishment" that "testing for HIV antibodies should be at the discretion of the patient's doctor and should not necessarily require the consent of the patient". Three anxious months later, we have leading Counsel's advice that any doctor acting upon this policy would be exposed, upon challenge in the courts, to the near certainty of both civil and criminal conviction. Unlike those tests for which consent is traditionally implied, such as WR or blood urea, testing for HIV antibodies cannot be regarded as routine or in the best interests of the patient. We did better to approve new manpower proposals for the NHS, to press for no-fault compensation for patients, for safeguards for confidentiality of personal health information and for the urgent study of the vexed problems of sexual abuse in children.

It was fitting to the shipshape Bristol organisation that the local Division should hold its summer reception on the last evening of the meeting on the S.S. Great Britain. As the sun set behind the yardarm, we toasted the past and the future. So to Norwich next year, with Bristol graduate David Pickersgill in charge of the local organising committee. And in 2007?

Sandy Macara

(Dr Macara was elected deputy chairman of the meeting, his 21st as a representative of the Bristol Division. Ed.)

**Care of the Elderly**
**News from the University Department**

The Dementia Research Unit is growing. We have a small clinical research team running a Memory Disorders Clinic and evaluating new approaches to treatment, including THA. The successful treatment of behavioural disorders with Trazodone and Tryptophan in moderately to severely demented patients, as described in our letter to the *Lancet* earlier this year, has freed many people from the side-effects of increasing doses of phenothiazines. It appears to help between 70 and 80% of aggressive, noisy, or otherwise disturbed sufferers with Alzheimer's disease.

The study undertaken by the small neuropathology research team into changes in subcortical nuclei has shown that not all subjects with senile Alzheimer's disease have a cholinergic deficit, either biochemically or in terms of cell loss in the basal nucleus of Meynert. This has important therapeutic implications.

Work under way in the neurotransmitter lab. in the B.R.I. indicates that the cell loss in those people in whom it occurs is probably not as great as was previously suspected. It rather looks as if many of the cells that were considered to have died are still present, albeit non-functioning. We are therefore currently investigating potential neurotrophic factors that may selectively stimulate regeneration in these cells, rather than trying to treat the disease by blanket neurotransmitter enhancement at a biochemical level. This is also important in terms of its therapeutic implications.

As in most University Department's, our research is being conducted on a shoestring, often also subject to the goodwill of others who are making resources and facilities available to us. In an attempt to provide some financial stability a small group of people have got together to try and raise an endowment sum to support the research activities of the Department. This has been christened "Bristol Research into Alzheimer's and Care of the Elderly" or "BRACE", and will be launched officially in September. We hope to use the resources raised to stabilize the funding for the research scope and encourage and foster research by other disciplines into the problems of older people. The need for additional accommodation has been resolved by the provision at Manor Park Hospital of a small clinical research unit for all the consultants working within the District. I would like to express our gratitude to our Administrator, Kevin Hogarty, and the Authority for their support with this.

As many people will know, Dr. Bill Lloyd has now retired from his post of Consultant Geriatrician with the Bristol & Weston Health Authority. He has become part of the history of Geriatric Medicine in Bristol, and there are many who owe a lot to his wise counsel and guidance. I'm sure that readers of the Journal will join with me in wishing him well in his retirement. In his place we would like to extend a warm welcome to Dr. Gerald Tobin, who has joined the Bristol & Weston team in Bill's place. He was appointed to the Bristol post after Senior Registrar training in Liverpool. We're all looking forward very much to working with him.

G. K. Wilcock

**"On the receiving end"**

My new routine was borne of an awareness of my coronary arteries. Papers in countless journals, advice from doctors and vegans alike and the gallows humour we use to defend our decadent habits had me pedalling my way towards a healthy future after a long day at work during which I had exercised nothing further south than my odontoid peg. Not the scientists, nor the faddists nor the cynically mirthful had warned me of the unseen danger of shunning our culture's habitual mechanised transport or of the paradoxical way one's intentions could backfire and transform one's rôle from carer to cared for.

And then quite simply my time had come; my number was up. I lay in the road noting how fast the world moves around you when you cannot move yourself. People run, lights flash, bells ring. From all sides people in uniforms appear as if by magic. Without anticipation I had embarked upon my first and I hope my only short sharp course in rehabilitation. You might call it a crash course.

Ambulancemen are superb. As an experienced doctor, how can I fail to recognise the characteristic signs of shock when they happen to me instead of to 'a patient'. How can I not realise that the faintness, sweating, and that feeling that I am about to have an accident of a much more personal nature, merely require a change of posture to be relieved instantly? If only the same miraculous cure could apply to that slow-motion nuclear explosion which seems to be going critical somewhere down in the distal region of my left leg.

Nobody seems to be interested in the fact that every vibration seems to trigger a re-enactment of Chernobyl within the confines of one of my limbs. Instead I am expected to do the impossible and simply lift myself from the tarmac to the stretcher that has appeared beside me. Every attempt precipitates nuclear war, not global, but local to my left ankle. Again the ambulancemen have the answer, in the way I presume they have had a thousand times previously. At first I wonder why they are playing with an inflatable model at a time like this. I thought such things were only found in sex shops (but maybe that is because I am a psychiatrist, or is there some other reason?). Then I realise; an inflatable trauma splint. Gentle tumescence supports my comminution. Maybe not a thrill or even subtle stimulation but at least something more neutral than explosively painful sensation. Two inches of air form an impregnable cushion from external insult. Surely this simple device must win the prize for the most cost-effective invention ever.

Initially I was aware of the Accident and Emergency Department around me. Then steadily the world shrank; or was it my left ankle growing? Either way, the pain mushroomed like a black cloud from the far end of the trolley on which I lay. All other modalities were obliterated as the stark blackness of pain darkened my entire sensorium. Previously I had thought I could endure pain. In my arrogant past I had insisted on having my skin sutured without local anaesthetic. I tell my dentist not to bother with an injection. Why should I have analgesia now? My principles make me refuse the profferred opiate. The nurses' competence make them insist and the needle enters my upper outer quadrant. "Sissy", I mutter to myself. I ponder upon the gate theory, on the higher levels of control of pain, on the influence of psychological set. I force a false chuckle through true agony and assert that analgesia only works if you want it to.

In due course I discover I was right all along. Morphine does not work. The pain is still there. Yet, undeniably, I was pleased with the result of the injection. The paradox engages my curiosity until I remember the relevant pharmacological text, "...sedation, euphoria and mental detachment may enhance the sense of well-being". Yes, the pain was still present, but a sense of well-being was also present and I did not seem to mind! I could look down and examine the ravages wrought by the holocaust at the other end of the trolley. I could even rate the pain: mild, moderate, severe? Yes, it was severe; and I could describe what made it better and what made it worse, where it was tender and where it was not; but I did not seem to mind. No longer did it seem a subjective experience; no longer part of me. Detached mentally, I could now cooperate with those people, the doctors and nurses, who only minutes ago seemed bent on pestering me, on attacking my very Achilles heel.

The revered pharmacology of the common poppy would have made my wait for operation enjoyable had I been able to quench my thirst. Why do we have so many drugs to cause a dry mouth but none able to cure it? I wanted to talk, to describe my new-found iatrogenic euphoria, but my lips stuck together, my tongue glued to each bit of gum it touched. I could not swallow. I would have preferred to suffer my pain rather than this new form of purgatory. Anaesthesia when it came was bliss.

Rehabilitation, they say in psychiatry, begins on the day of admission. So too it seems in orthopaedics. I wake to find a girl in white assaulting my foot, my red, swollen, throbbing foot which only hours ago was laid bare to the bone on an operating table. Yet vaguely I now recall the orthopaedic text, "The advantages of internal fixation are early mobilisation". For me cognitive dissonance surrounds the concept that something so painful can be an advantage. But to this day I am mobile.

In a subtle way the most challenging stage was half way to recovery. Some useful function had returned to my injured limb. With an effort I could do things for myself. With a request I could have things done for me. How tempting to feign that extra bit of disability and have things done for me. What a disincentive to recovery. I learned that I could swing the lead as skillfully as any patient – after all, in my time I have unwittingly colluded with simulation far more blatant than this. Why should people not collude with me now that the tables are turned? How gullible are friends and loved ones in their eagerness to help. They gave me grace. They sanctioned my sickness.

But my greatest tribute goes to the staff, to those highly trained professionals who knew when not to help. Disregard of the psychological when attending to the physical is freqently criticised, but my view at least has changed. Their psychology was to disregard mine, and I hated them for it. But the point when they made their decision not to help was every bit as important as their action to help had been a fortnight earlier. They were right now just as they were right then. I could walk. I do walk.

Anthony White

**Five Icons for St. Peter**

The year 1988 will mark the 10th anniversary of the appointment of the first home care nurse in the service of St. Peter's Hospice. The Friends of St Peter's are planning a number of special occasions to mark this event. One of these is to be held at St. George's Brandon Hill, Bristol on Wednesday, 10 February, 1988 and will include the first performance of a violin sonata by the Bristol composer, Nigel Dodd, by the brilliant young violinist, Ishani Bhoola. Ishani, who is the daughter of Dr Kanti Bhoola, Reader in Pharmacology in the University of Bristol, has a whole string of scholarships to her credit. She is currently in her fourth year of study with Erich Gruenberg at the Guildhall School of Music. The pianist will be Pamela Lidiard who has worked as an accompanist at the National Opera Studio and the Britten-Pears School at Snape, she has made several broadcasts and is establishing a reputation as a recitalist.

Nigel Dodd told me that the work which is entitled *Five Icons for St. Peter* is built around various aspects of the character of St Peter and events in his life. The five movements are entitled *The Miraculous Draught of Fishes, The Impulsive Peter, Peter the Visionary, The Tears of St Peter* and *Cloven Tongues as of Fire*. The whole idea grew from a request from Ishani's parents that he would write a work for her 21st birthday. The idea of associating it with St Peter seemed to present itself as an analogy with Messiaen's *Vingt Regards*, and Dr Bhoola has been a strong supporter of the Hospice since its inception.

Recent works by Nigel Dodd include *The Ascension*, a cantata (May 1986), *The Rainbow Cantata* (Nov 1986) and *The Bristol Tryptich* for piano and string orchestra (March 1987). He also wrote the *Southmead Idyll* for solo viola, harp and orchestra in June 1985, to celebrate the 21st birthday of the Southmead Hospital Orchestra – an analogy with Wagner's *Siegfried Idyll* written to celebrate the birth of his son. Nigel Dodd studied composition with Edmund Rubbra, his music is immediately recognisable as in the English tradition, it is melodic and inventive, he can write fine tunes and it has a direct appeal. The audience at St George's on February 10th will not only be helping St Peter's and hearing the first performance of a new sonata by the very talented daughter of a Bristol doctor, but they are also sure tc enjoy themselves. The programme will also include works by Beethoven, Tchaikovsky and Saint Saens.

M. G. Wilson

